# Magnitude and risk factors of mother-to-child transmission of HIV among HIV-exposed infants after Option B+ implementation in Ethiopia: a systematic review and meta-analysis

**DOI:** 10.1186/s12981-024-00623-6

**Published:** 2024-06-07

**Authors:** Wolde Facha, Takele Tadesse, Eskinder Wolka, Ayalew Astatkie

**Affiliations:** 1 Department of Epidemiology and Biostatistics, School of Public Health, College of Health Sciences and Medicine, Wolaita Sodo University, Wolaita Sodo, P.O.BOX 136, Wolaita Sodo, Ethiopia; 2 School of Public Health, College of Medicine and Health Sciences, Hawassa University, P.O.BOX: 1560, Hawassa, Ethiopia

**Keywords:** Mother-to-child transmission, MTCT, PMTCT, HIV-exposed infant, Systematic review, Meta-analysis, Ethiopia

## Abstract

**Background:**

Mother-to-child transmission (MTCT) of the human immunodeficiency virus (HIV) remains a major public health challenge in Ethiopia. The objective of this review was to assess the pooled magnitude of MTCT of HIV and its risk factors among mother-infant pairs who initiated antiretroviral therapy (ART) after Option B+ in Ethiopia.

**Methods:**

A systematic search of literature from PubMed, Hinari, African Journals Online (AJOL), Science Direct, and Google Scholar databases was conducted from June 11, 2013 to August 1, 2023. The authors used the Preferred Reporting Items for Systematic Reviews and Meta-Analysis (PRISMA) guidelines to guide the article selection process and reporting. Observational studies that reported the magnitude and/or risk factors on MTCT of HIV among mother-infant pairs who initiated ART after the implementation of Option B+ in Ethiopia were included. We applied a random-effect model meta-analysis to estimate the overall pooled magnitude and risk factors of MTCT of HIV. A funnel plot and Egger’s regression test were employed to check publication bias, and heterogeneity was assessed using I^2^ statistics. The protocol was registered in the PROSPERO database with registration ID number CRD42022325938.

**Result:**

Eighteen published articles on the magnitude of MTCT and 16 published articles on its risk factors were included in this review. The pooled magnitude of MTCT of HIV after the Option B+ program in Ethiopia was 4.05% (95% CI 3.09, 5.01). Mothers who delivered their infants at home [OR: 9.74; (95% CI: 6.89–13.77)], had not been on ART intervention [OR: 19.39; (95% CI: 3.91–96.18)], had poor adherence to ART [OR: 7.47; (95% CI: 3.40–16.45)], initiated ART during pregnancy [OR: 5.09; (95% CI: 1.73–14.97)], had WHO clinical stage 2 and above [OR: 4.95; (95% CI: 1.65–14.88]], had a CD4 count below 350 at enrolment [OR: 5.78; (95% CI: 1.97–16.98], had no or low male partner involvement [OR: 5.92; (95% CI: 3.61–9.71]] and whose partner was not on ART [OR: 8.08; (95% CI: 3.27–19.93]] had higher odds of transmitting HIV to their infants than their counterparts.

**Conclusion:**

This review showed that the pooled magnitude of MTCT of HIV among mother-infant pairs who initiated ART after the Option B + program in Ethiopia is at the desired target of the WHO, which is less than 5% in breastfeeding women. Home delivery, lack of male partner involvement, advanced HIV-related disease, lack of PMTCT intervention, and poor ARV adherence were significant risk factors for MTCT of HIV in Ethiopia.

**Supplementary Information:**

The online version contains supplementary material available at 10.1186/s12981-024-00623-6.

## Background

Human immunodeficiency virus (HIV) continues to be a major global public health threat, particularly in low- and middle-income countries (LMICs), including Ethiopia [1, 2]. Globally, nearly 39 million people are living with HIV, 53% of whom are women and girls [3]. About 1.3 million people were newly infected with HIV, and 130,000 of these infections occurred among children in 2022 [3, 4]. Over 63% of newly infected HIV patients live in sub-Saharan Africa, which makes the region the most affected on the continent [5]. About 12,000 new infections occurred in Ethiopia in 2020 [6]. Most of these new infections are due to MTCT, which accounts for 90% of new infections [7]. Without any intervention, about 20–45% of breastfeeding children acquire HIV infection from their mothers [8].

To reverse the burden, Ethiopia has been implementing the World Health Organization’s (WHO) Option A and Option B recommendations for the prevention of mother-to-child transmission (PMTCT) of HIV since December 2011 [9–11]. These options involved the use of an antiretroviral drug(s) for prophylactic and treatment purposes based on WHO clinical stage and immunologic status. Despite the efforts to rapidly reduce and subsequently eliminate MTCT among HIV-exposed infants by revising treatment protocols, the vision of an HIV-free new generation target by 2020 was not achieved in the country [12, 13]. In this regard, Ethiopia adopted a third option (Option B+) as the preferred strategy for the PMTCT of HIV in 2013 [14]. Option B+ consists of lifelong ART for all HIV-infected pregnant and lactating women (PLW), irrespective of their CD4 count and WHO clinical staging [8].

The Joint United Nations Programme on HIV/AIDS (UNAIDS) targeted zero new infections among infants born to HIV-positive women by 2020 [15]. Ethiopia has also developed two strategic plans, the first from 2013 to 2015 and the second from 2017 to 2020, to achieve a transmission rate of HIV of less than 2% in non-breastfed infants and less than 5% in breastfed infants [5, 16]. However, different reviews showed that the pooled magnitude of MTCT for HIV in Ethiopia ranged from 5.64 to 11.4% [17–20]. The previous systematic review and meta-analysis conducted in Ethiopia used studies that included mother-infant pairs enrolled in care before and after implementing the Option B + program. This could not indicate the existing pooled magnitude of MTCT for HIV since the intervention approach (eligibility criteria to initiate ART for mothers and duration of prophylaxis for infants) was different before and after Option B+. Therefore, this review is conducted to answer the following two questions: (1) What is the pooled magnitude of the MTCT of HIV in Ethiopia after the implementation of Option B+? and (2) What are the risk factors for MTCT of HIV in Ethiopia after the implementation of Option B+?

## Methods

### Study design and reporting

The design of this review was a systematic review and meta-analysis, and the protocol was registered in the PROSPERO database with registration ID number CRD42022325938. The Preferred Reporting Items for Systematic Reviews and Meta-analysis (PRISMA) 2020 statement guideline was followed to report the results [21].

### Database and search strategy

The authors used PubMed, Hinari, AJOL, Science Direct, and Google Scholar electronic databases. The PRISMA 2020 guideline was implemented to guide the article selection process and reporting. Studies were searched using the title, keywords, and MeSH terms from databases. The following keywords were used to search the articles in the database: “MTCT”; “Mother-to-Child Transmission”; “PMTCT”; “Prevention of Mother-to-Child Transmission”; “Vertical transmission”; “HIV transmission”; “HIV”; “Human immunodeficiency virus”; “Option B+”; “Option B plus”; “HIV Testing”; “HIV Seroprevalence”; “HIV Seropositivity”; “HIV Infections”; “Exposed infant”; “HIV-exposed infant”; “Infant”; “Associated factors”; “Risk factors”; “Determinants”; “Predictors”; “Prevalence”; “Incidence”; and “Ethiopia”. The search string was developed using “AND” and “OR” Boolean operators. An example of the search details for PubMed is provided in Additional File 1. Besides, gray literature and reference lists of relevant articles were also retrieved to find additional studies. Journal articles conducted after the implementation of Option B + in Ethiopia in the 10 years preceding the review were included in the review. Mendeley software was used to remove duplicate references and for citation purposes.

### Eligibility criteria

Observational studies (cross-sectional, case-control, and cohort studies) that reported prevalence and/or risk factors for MTCT of HIV and were conducted from June 11, 2013 to August 01, 2023 were included in the present review. Besides, the review included articles that were conducted among mother-infant pairs enrolled in PMTCT care only after the implementation of Option B + in Ethiopia and published in the English language. Studies without the outcome of interest, conducted before the implementation of Option B + program, with only abstracts, qualitative studies, a JBI score below 50%, and citations without full text were excluded.

### Variables

The outcome variable in this review was HIV infection status among infants born to HIV-positive women. The HIV infection status was determined at 6 weeks of age or earliest health care contact then after using DNA/PCR virology tests, or at the age of 18 months or older using rapid antibody (serological) tests after 6 weeks of breastfeeding cessation [22]. An infant with a positive test result for either DNA/PCR or antibody was said to be HIV-infected [8, 22]. Maternal and infant-related variables associated significantly with the outcome of interest (HIV infection status) among exposed infants were abstracted from independent studies to determine risk factors for MTCT of HIV. In this review, place of delivery, ANC follow-up, timing of ART initiation, WHO clinical stage, CD4 count, male partner involvement, partner HIV status, and adherence to ART were maternal-related covariates, while adherence to nevirapine (NVP) prophylaxis, feeding practice, and age at enrolment were infant-related covariates. Maternal ART adherence and infant NVP adherence were measured according to missed doses out of 60 doses. It was said to be good if they missed less than or equal to 3 doses; fair if they missed 4–9 doses; and poor if they missed greater than or equal to 10 doses out of 60 doses [8, 23]. The level of male partner involvement in the PMTCT program was measured using six questions. These were whether the male partner attended ANC with his partner, knew the partner’s antenatal appointment, discussed antenatal interventions with his partner, supported his partner’s antenatal visits financially, sought permission to use a condom during the current pregnancy, and tested for HIV with his partner or not. A total score of four to six was considered high partner involvement, and less than four was considered low partner involvement [24, 25].

### Quality assessment

All retrieved studies were exported to the Mendeley software reference manager, and duplicate studies were removed carefully. All authors agreed on the type of databases, keywords, MeSH terms, Boolean operators, eligibility criteria, and data extraction template before the review process. The authors screened the articles based on titles and abstracts, then thoroughly reviewed full-text articles to be included in the review as per the eligibility criteria and extracted the data for analysis. The quality of articles included in the study was evaluated using Joanna Briggs Institute (JBI) quality appraisal criteria adapted for prevalence, cohort, and case-control studies [26, 27]. A Joanna Briggs Institute (JBI) score value of 50% and above was considered to ensure the quality of selected articles included in the review (Additional File 2).

### Data extraction

The authors extracted data from selected articles using the data extraction templates that include the first author, publication year, study design, study region, study area, response rate, sample size, event (number of newborns positive for HIV), and JBI score for the prevalence study (Additional File 3). Data for risk factors for the MTCT of HIV infection were collected from cell values in two-by-two tables for exposure and outcome variables using Microsoft Excel.

### Data analysis

Abstracted data were saved in Microsoft Excel (MS Office 2010) and then exported to Stata 14 (Stata Corp., College Station, TX, USA) statistical software to perform a meta-analysis. A random-effects model using the Stata command ‘metan’ was used to estimate the pooled magnitude and risk factors of HIV infection due to MTCT. Risk factors that have a significant association with the MTCT of HIV were reported using a pooled OR with a 95% CI. Forest plots were used to present the pooled magnitude of HIV infection with its 95% CI. Subgroup analysis was performed by study region and study design to show the variations in the pooled estimate of the magnitude. The percentage of total variation across studies due to heterogeneity was assessed using the I^2^ statistic along with the corresponding p-value. A p-value less than 0.05 from Cochrane’s Q test indicated significant heterogeneity. I^2^ statistics of 25% were considered as low, 50% as moderate, and 75% as high heterogeneity. Publication bias across studies was checked using a funnel plot. Egger’s regression test was used to evaluate publication bias; *p* < 0.05 declared statistically significant bias in publication.

## Results

### Search results

**Fig. 1:**
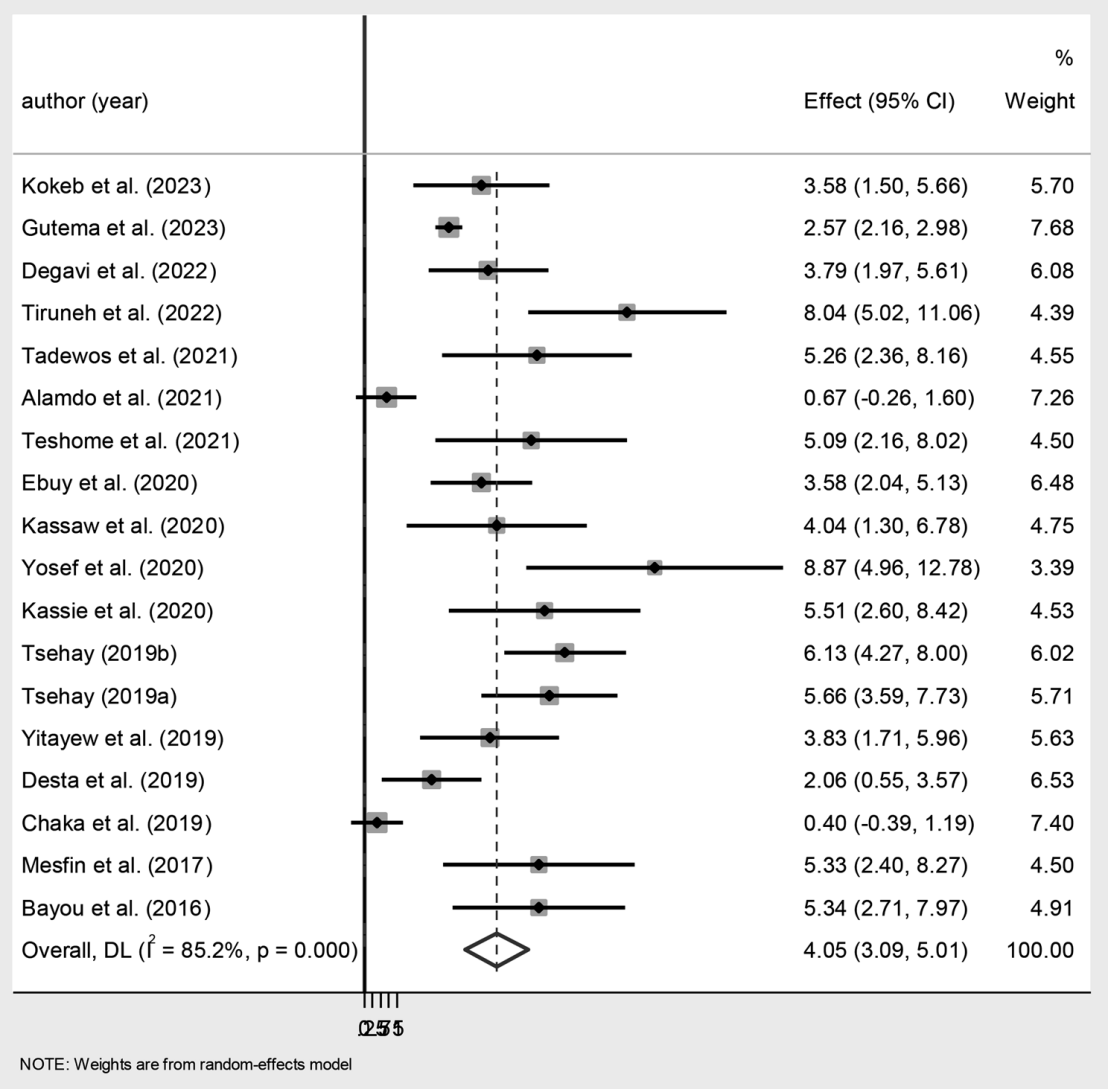
Forest plot of the magnitude of MTCT of HIV after Option B + in Ethiopia. Dots represent the magnitude reports with confidence intervals (CIs) from primary studies. The diamond shape represents the pooled estimation, and the width of the diamond shows the width of the 95% CI of the pooled estimate

The authors found a total of 1,437 published articles issued in the ten years preceding the review from different sources before the removal of duplicated articles. These articles were obtained from PubMed (*n* = 533), Hinari (*n* = 386), AJOL (*n* = 41), Science Direct (*n* = 374), Google Scholar (*n* = 81), and grey literature search (*n* = 22). The authors conducted a preliminary screening that yielded 109 articles for further screening based on full text. A total of 18 articles (ten cross-sectional and eight cohorts) were included to get the pooled magnitude of the MTCT of HIV, whereas 16 articles (eight cross-sectional, four cohort, and four case-control) were included to identify risk factors. Figure 1 shows the entire process of study screening and selection.

### Study characteristics

This review consists of ten cross-sectional studies [28–37] that involved 8,939 HIV-exposed infants, and eight cohort studies [38–45] that involved 2,237 HIV-exposed infants to calculate the pooled magnitude of the MTCT of HIV (Table 1). The smallest and largest sample sizes were 96 [46] and 5,679 [28], respectively. Based on the JBI quality appraisal criteria, all studies fulfilled the minimum quality criteria.

**Table 1:** Characteristics of the original studies included in a meta-analysis of the magnitude of MTCT of HIV after Option B + in Ethiopia

Author(Year)	Study design	Study region	Sample size	Event*	Response rate	JBI score (%)
Kokeb et al. (2023)	Cohort	Amhara	307	11	95.34	73
Gutema et al. (2023)	Cross-sectional	Ethiopia	5,679	146	83.1	89
Degavi et al. (2022)	Cohort	Oromia	422	16	91.3	64
Tiruneh et al. (2022)	Cohort	Amhara	311	25	100	73
Tadewos et al. (2021)	Cohort	SNNP**	228	12	100	82
Alamdo et al. (2021)	Cohort	Addis Ababa	298	2	83.7	73
Teshome et al. (2021)	Cross-sectional	Addis Ababa	216	11	100	89
Ebuy et al. (2020)	Cross-sectional	Tigray	558	20	70	67
Kassaw et al. (2020)	Cohort	Amhara	198	8	91.2	73
Yosef et al. (2020)	Cross-sectional	SNNPR**	203	18	100	78
Kassie et al. (2020)	Cross-sectional	Amhara	236	13	98.8	89
Tsehay (2019b)	Cross-sectional	Amhara	636	39	100	89
Tsehay (2019a)	Cross-sectional	Amhara	477	27	100	89
Yitayew et al. (2019)	Cross-sectional	Amhara	313	12	90.2	89
Desta et al. (2019)	Cross-sectional	Tigray	340	7	97.1	67
Chaka et al. (2019)	Cohort	Oromia	248	1	98.7	55
Mesfin et al. (2017)	Cohort	Oromia	225	12	100	55
Bayou et al. (2016)	Cross-sectional	Oromia	281	15	92.8	78

*Event Number of HIV-infected children among HIV-exposed infants; ** Southern Nations, Nationalities, and People’s Region

### Meta-analysis

#### Magnitude of MTCT of HIV

The meta-analysis showed that the pooled magnitude of the MTCT of HIV in Ethiopia was 4.05% (95% CI 3.09, 5.01) (Fig. 1).

### Subgroup analysis

**Fig. 2:**
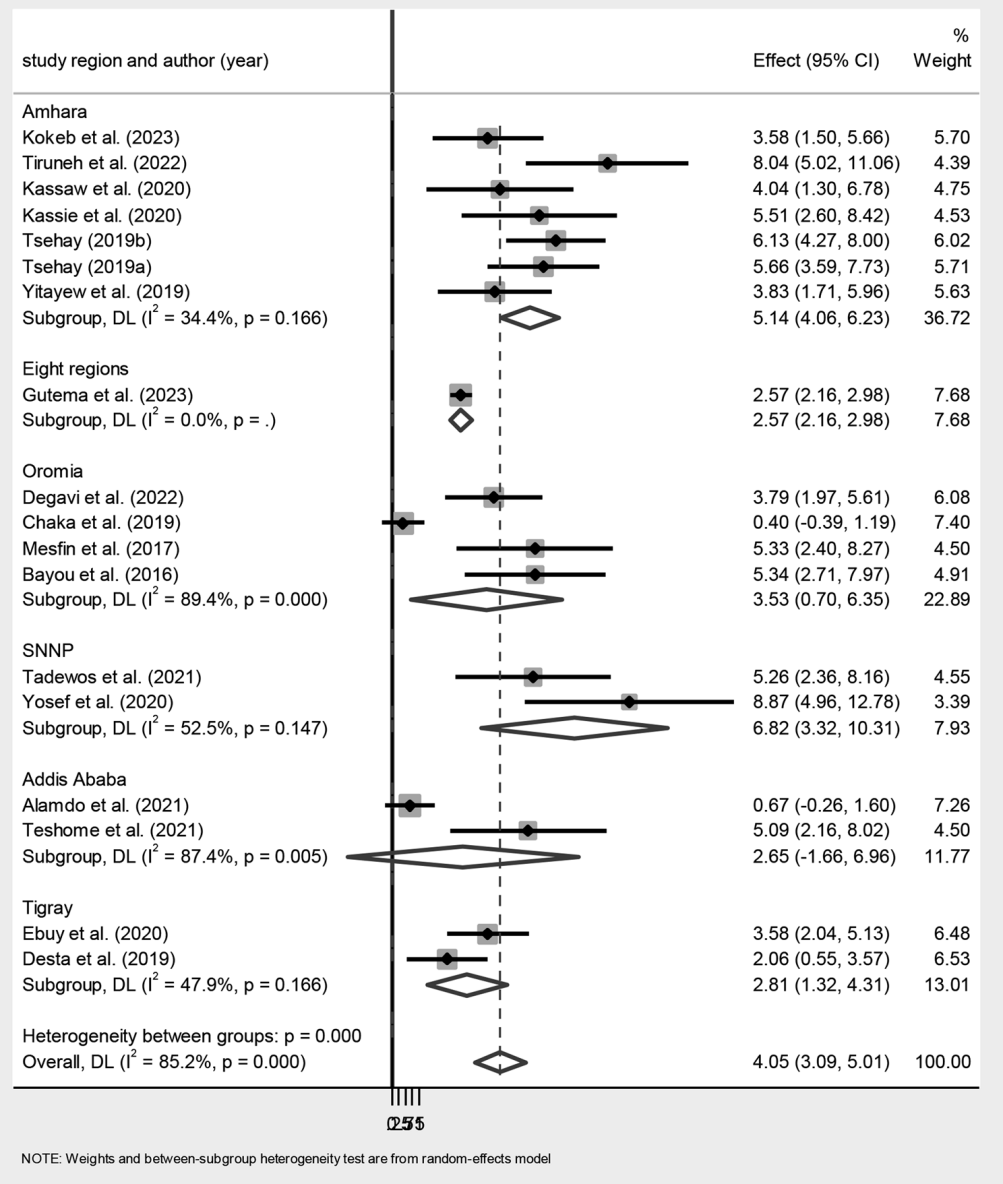
Subgroup analysis of the magnitude of MTCT of HIV after Option B + in Ethiopia by study region. Dots represent the magnitude with 95% confidence intervals from primary studies, and the diamond shape stands for the pooled estimation

Due to heterogeneity across the studies (I^2^ = 85.2%, p-value < 0.001), subgroup analysis was carried out with the study region and study design. The subgroup analysis by study region showed that the magnitude of MTCT of HIV was highest in the SNNP region [6.82% (95% CI: 3.32, 10.31)] and lowest in studies conducted by the Ethiopian Public Health Institute (EPHI) from eight regions [2.57% (95% CI: 2.16, 2.98)]. (Fig. 2).

**Fig. 3:**
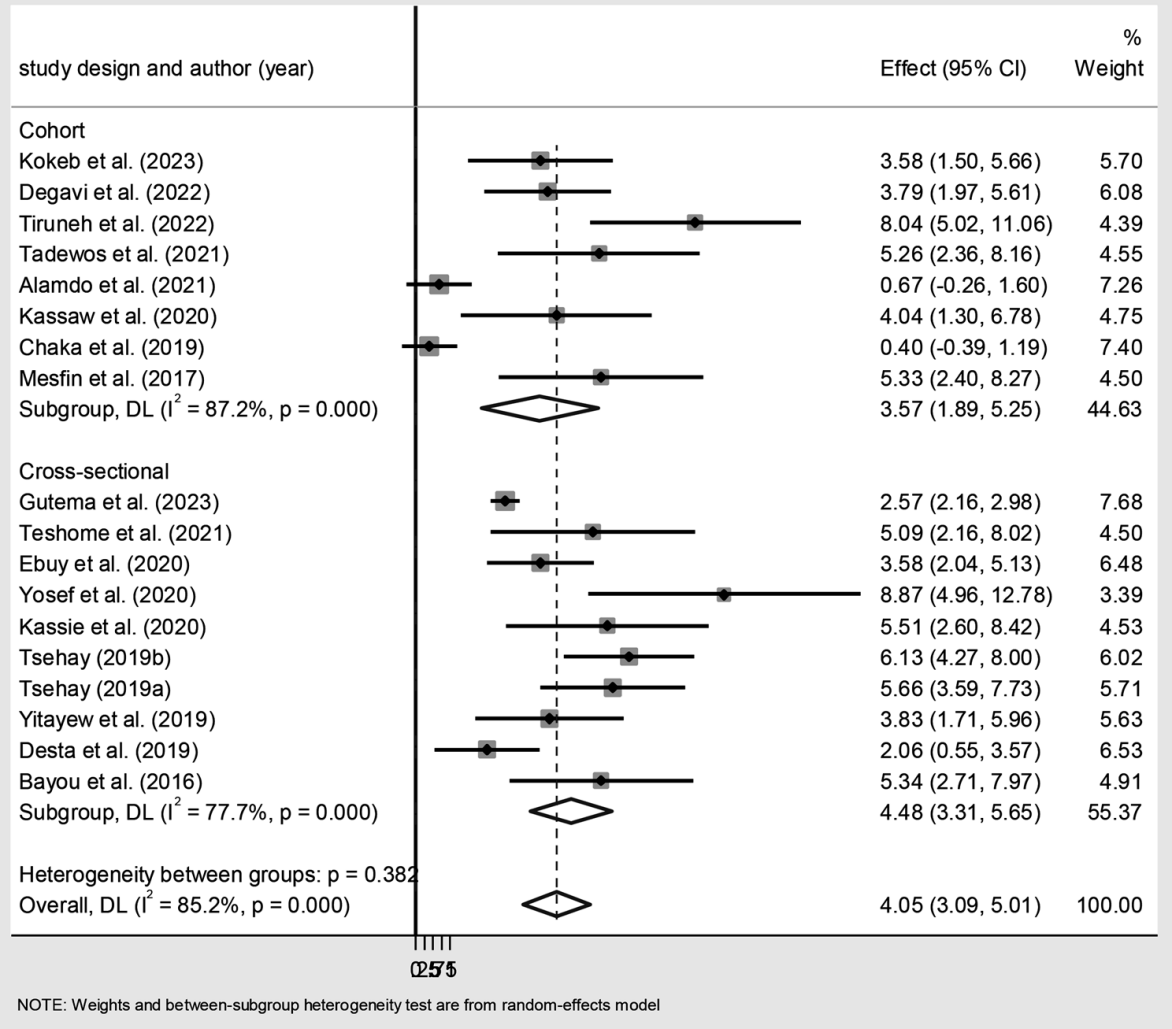
Subgroup analysis on the magnitude of MTCT of HIV after Option B + in Ethiopia by study design. Dots represent the magnitude reports with confidence intervals from primary studies, and the diamond shape stands for the pooled estimation

The subgroup analysis by study design showed that the pooled magnitude was 3.57% (95% CI: 1.89, 5.25) for the cohort studies and 4.48% (95% CI: 3.31, 5.65) for the cross-sectional studies (Fig. 3).

### Factors associated with the MTCT of HIV

This review consists of eight cross-sectional studies [28–30, 32–35, 37] that involved 8,100 HIV-exposed infants, four cohort studies [40, 43, 44, 47] that involved 1,159 HIV-exposed infants, and four case-control studies [23, 25, 46, 48] that involved 926 HIV-exposed infants to assess risk factors for the MTCT of HIV. The smallest and largest sample sizes were 96 [46] and 5,679 [28] respectively. Based on the JBI quality appraisal criteria, all studies included in the meta-analysis of risk factors for MTCT of HIV fulfilled the minimum quality criteria (Table 2).

**Table 2:** Characteristics of the original studies included in the meta-analysis of the risk factors for MTCT of HIV after Option B + in Ethiopia

Author(year)	Study design	Study region	Sample size	Infant’s HIV status		JBI score (%)
				Positive	Negative	
Alemu et al. (2022)	Case-control	Amhara	320	66	254	70
Beyene et al. (2018)	Case-control	AA*	220	44	176	80
Hunduma et al. (2021)	Case-control	Oromia	96	24	72	70
Hussen et al. (2022)	Case-control	SNNP**	290	58	232	80
Desta et al. (2019)	Cross-sectional	Tigray	340	7	333	67
Gutema et al. (2023)	Cross-sectional	Ethiopia	5,679	146	5533	89
Kassie et al. (2020)	Cross-sectional	Amhara	236	13	223	89
Teshome et al. (2021)	Cross-sectional	AA*	216	11	205	89
Tsehay(2019b)	Cross-sectional	Amhara	636	39	597	89
Tsehay(2019a)	Cross-sectional	Amhara	477	27	450	89
Yitayew et al. (2019)	Cross-sectional	Amhara	313	12	301	89
Yosef et al. (2020)	Cross-sectional	SNNP	203	18	185	78
Degavi et al. (2022)	Cohort	Oromia	422	16	406	64
Kassaw et al. (2020)	Cohort	Amhara	198	8	190	73
Tadewos et al. (2021)	Cohort	SNNP**	228	12	216	82
Tiruneh et al. (2022)	Cohort	Amhara	311	25	286	73

*Addis Ababa, **Southern Nations, Nationalities, and People

Based on the review, risk factors for the MTCT of HIV were categorized into four thematic areas. These were: obstetric, drug and clinical, male partner-related, and infant-related factors (Table 3). The pooled OR for each risk factor is mentioned below (Additional File 4).

### Obstetric factors

Mothers who delivered their infants at home [OR: 9.74; (95% CI: 6.89–13.77)] had higher odds of transmitting HIV to their infants than their counterparts (Additional File 5).

### Drug and clinical-related factors

Mothers who had not been on ART intervention [OR: 19.39; (95% CI: 3.91–96.18)], had poor adherence to ART [OR: 7.47; (95% CI: 3.39–16.45)], initiated ART during pregnancy [OR: 5.09; (95% CI: 1.73–14.97)], had WHO clinical stage 2 and above [OR: 4.95; (95% CI: 1.65–14.88)], and had a CD4 count below 350 at enrolment [OR: 5.78; (95% CI: 1.97–16.98] had higher odds of transmitting HIV to their infants than their counterparts.

### Male partners-related factors

Mothers who had no or low male partner involvement [OR: 5.92; (95% CI: 3.61–9.71)] and whose partner was not on ART [OR: 8.08; (95% CI: 3.27–19.93)] had higher odds of transmitting HIV to their infants than their counterparts.

### Infant-related factors

Infants who had mixed feeding practice [OR: 10.66; (95% CI: 4.12–27.54)], enrolled in ART care lately (> 6 weeks) [OR: 6.97; (95% CI: 3.22–15.11)], had not received nevirapine prophylaxis [OR: 17.79; (95% CI: 7.67–41.27)], had poor adherence to nevirapine [OR: 15.82; (95% CI: 5.0–50.10)], and were undernourished [OR: 8.94; (95% CI: 4.53–17.66] had higher odds to acquire HIV from their mothers than their counterparts.

**Table 3:** Study characteristics included in a meta-analysis of the risk factors for MTCT of HIV after Option B+ in Ethiopia

Variable	Number of studies	Sample size	Effect size (95% CI)	(I^2^, *P* value)
**Obstetric factorsF**				
Home delivery	8	2,550	9.74 (6.89, 13.77)	(16.0%, 0.304)
**Maternal drugs and clinical-related factors**				
No ARV intervention	3	749	19.39 (3.91, 96.18)	(71.4%, 0.03)
Poor ART adherence	4	1,033	7.47(3.39, 16.45)	(75.0%, 0.007)
Started ART during pregnancy	6	6,855	5.09(1.73, 14.97)	(87.1%, 0.001)
WHO stage 2 and above	6	2,028	4.95 (1.65, 14.88)	(89.9%,0.001)
CD4 below 350 count/mm3	3	655	5.78 (1.97, 16.98)	(29.1%, 0.244)
**Male partner-related factors**				
No or low involvement	3	606	5.92 (3.61, 9.71)	(12.4%, 0.319)
Not on ART	2	459	8.08 (3.27, 19.93)	(0.0%, 0.589)
**Infant-related factors**				
Mixed feeding practice	5	1,889	10.66 (4.12, 27.54)	(79.9%, 0.001)
Age > 6 weeks at enrolment	4	6,712	6.97 (3.22, 15.11)	(81.2%, 0.001)
No NVP prophylaxis	7	7,892	17.79 (7.67, 41.27)	(81.7%, 0.001)
Poor NVP adherence	2	743	15.82 (5.0, 50.10)	(66.3%,0.085)

### Publication bias

**Fig. 4:**
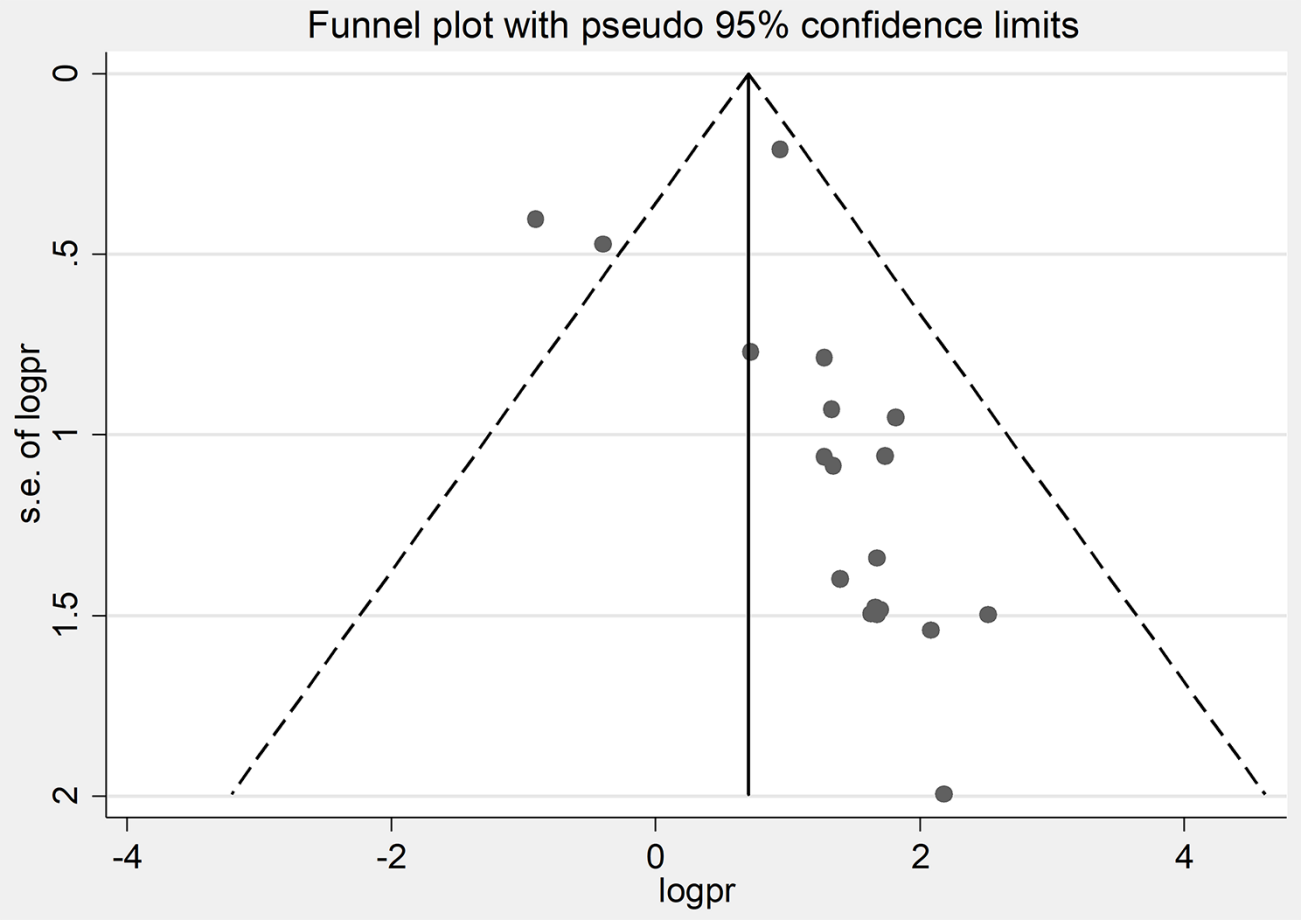
Funnel plot for the magnitude of MTCT of HIV after Option B+ in Ethiopia logpr, logarithm of the prevalence; se, standard error of the logarithm of prevalence.

A funnel plot showed that major studies fall within the predicted confidence interval except for two studies [39, 41] (Fig. 4). Though there was an asymmetrical distribution of the published articles, there were no outlier studies that influenced the overall effect size. Eggers’ regression test for funnel plot asymmetry revealed there is no significant publication bias (*p =* 0.088**).**

## Discussion

This study assessed the pooled magnitude of MTCT of HIV and its determinants among women who attended PMTCT services in Ethiopia. The review found that the pooled magnitude of MTCT of HIV was 4.05%. The finding is higher than the review conducted in Burkina Faso (0%), China (2.3%), and the global plan designed to achieve zero new infections among infants born to HIV-positive women by 2020 [15, 49, 50]. However, it is lower than the reviews conducted in East Africa, which was 7.68% [51], and in Ethiopia, which were 9.93% [18] and 11.4% [17]. The variation might be attributed to the differences in the study period and treatment protocol. The previous reviews included study subjects enrolled in PMTCT care before the implementation of the Option B + program, when the eligibility criteria to initiate antiretroviral drugs were different. This could highlight the effectiveness of the Option B + program in reducing MTCT rates compared to the previous Option A and Option B programs. It is also lower than the result of the review conducted in India, which was 8.76% [52]. The difference might be due to the socio-demographic, economic, healthcare coverage, or health-seeking behaviors of the populations in India and Ethiopia. The review showed that after the intervention of Option B+ (test and treat strategy), Ethiopia achieved the national target for reducing the MTCT rate of HIV to below 5% in breastfed infants [5, 16].

**Fig. 5:**
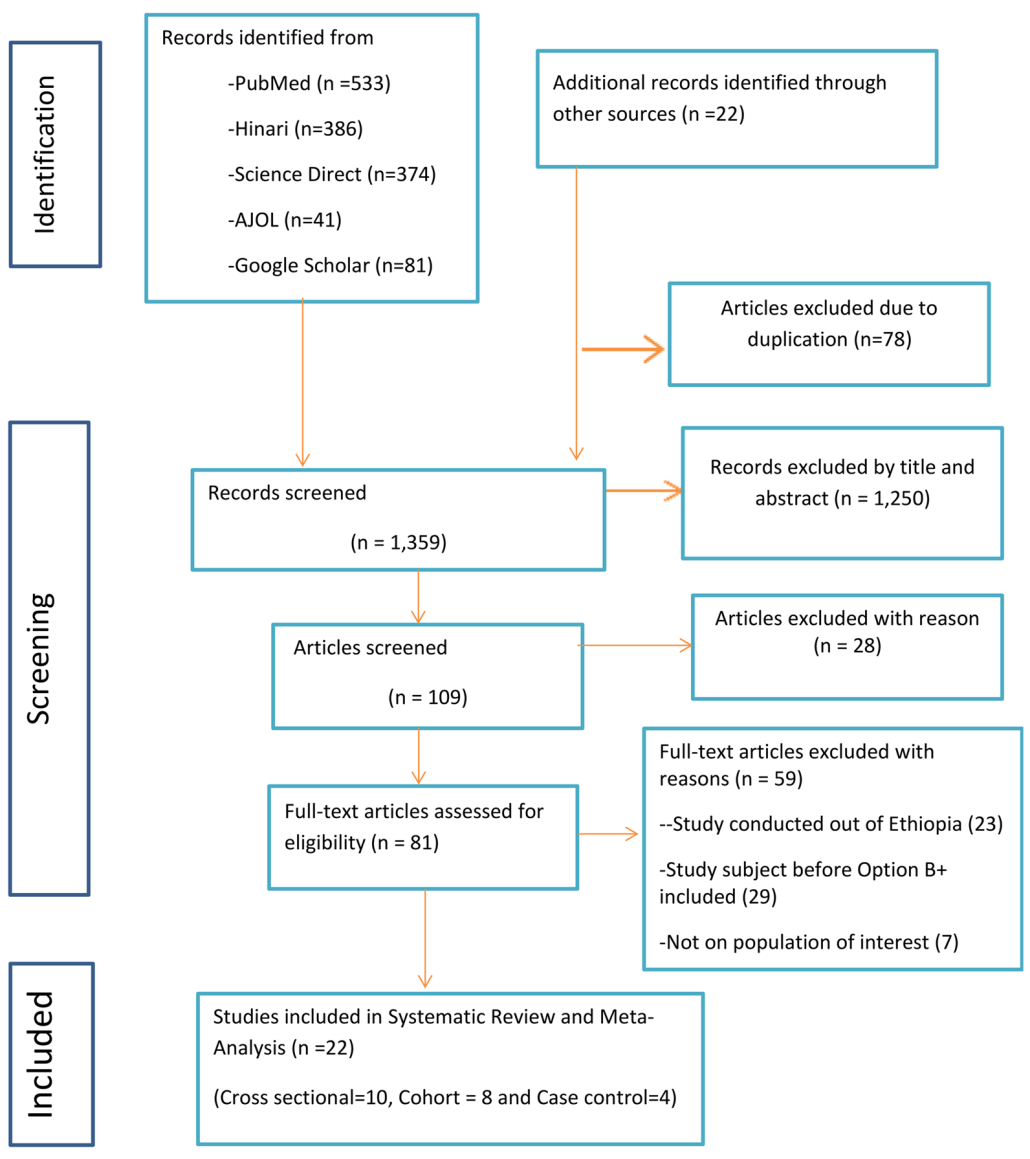
PRISMA 2020 flow diagram for studies included in the review, 2023

This review revealed that obstetric, drug and clinical, male partner, and infant-related factors were found to be the predictors of MTCT of HIV. In this review, home delivery was a determinant factor for the MTCT of HIV in Ethiopia. In this regard, those seropositive mothers who practiced home delivery had about ten times higher odds of transmitting the virus to their infants than those who delivered at a health facility. This finding is consistent with the review conducted in Ethiopia [17, 18, 51]. This could be due to the absence of safe delivery practices and other PMTCT interventions by mothers who gave birth at home, in which up to a third of HIV infection in infants occurs during labor and delivery [10]. In addition, those infants born at home missed the advantages of using ARV prophylaxes given immediately after birth that protect vertical transmission of HIV [8].

This review showed that those women who had not been on ART intervention at all had about ten times higher odds of transmitting HIV to their infants than those who were on ART. The finding is consistent with the review conducted in India and Ethiopia, which found that lack of maternal PMTCT intervention during the current pregnancy was a risk factor for MTCT of HIV [17, 18, 35, 51, 53]. The result also showed that mothers who initiated ART during pregnancy had about five times higher odds of transmitting HIV to their infants than those who started ART before pregnancy. Late initiation of ART could not give the full benefits of the drugs by protecting infants from acquiring HIV from their mothers [7, 16]. The finding is consistent with the review conducted in Ethiopia, where mothers who became pregnant after they knew their HIV positivity had higher odds of transmitting HIV to their infants [48]. This could be because those women who started ART before pregnancy and at least one month before delivery had reached viral suppression status and thus had a lower risk of transmitting HIV to their infants than those who started ART during labor, delivery, and then after [9].

According to this review, poor maternal adherence to ART results in about seven times higher odds of transmitting HIV to exposed infants, and poor infant adherence to nevirapine leads to about sixteen times higher odds of acquiring HIV from their mothers. This indicates that adherence to antiretroviral drugs among women and prophylaxis among exposed infants play a great role in the prevention of MTCT of HIV among exposed infants [8]. Those mothers who did not adhere to lifelong triple ART put their infants at higher risk of acquiring the virus than those mothers with good adherence [5, 14]. This could be because non-adherence to ART raises the risk of rapid viral replication, accelerates maternal HIV disease progression, and increases the possibility of MTCT of HIV [8]. Thus, adherence counselling should be provided at all antenatal care and postnatal visits to ensure that viral suppression is maintained throughout pregnancy and breastfeeding [5].

This review showed that mothers who had WHO clinical stage 2 or above had about five times higher odds of transmitting the virus to their infants than their counterparts. Besides, mothers whose CD4 count was below 350 cells/mm3 at enrollment had about six times higher odds of transmitting the virus to their infants than their counterparts. In the advanced clinical stage, there were more opportunistic infections (OIs) due to impaired immunity than those who were clinically stable (WHO clinical stage 1) [54]. The higher episodes of OIs coupled with the destroying capability of CD4-presenting T-cells by the virus lower the CD4 count [54, 55]. As a result, there were high viral copies that increased the risk of MTCT of HIV.

This review revealed that a lack of male partner involvement in HIV care and treatment was a risk factor for the MTCT of HIV. Thus, mothers who had no or low male partner involvement in care and treatment had about six times higher odds of transmitting HIV to their infants than their counterparts. The finding is supported by a review conducted in sub-Saharan Africa, which found that male partner involvement in the PMTCT program reduces the MTCT of HIV among exposed infants [50, 56, 57]. The study also showed that those mothers whose partners were not on ART had about eight times higher odds of transmitting HIV to their infants than their counterparts. This could be because the lack of financial, emotional, and psychological support from male partners made women not adhere to ART to get the benefits of medications for preventing MTCT of HIV [58–60].

The review found that infants who were on mixed feeding before the age of six months had ten times higher odds of acquiring HIV infection than infants who were on exclusive breastfeeding. The finding is in line with the review conducted in Ethiopia [17, 18, 51]. Mixed feeding during the first six months of life is believed to be a risk factor for the MTCT of HIV [5]. This could be because mixed feeding increases the incidence of gastrointestinal infections and ulceration secondary to diarrheal disease [61]. As a result, the virus can easily enter the infant’s bloodstream through ulcerated gastrointestinal tissue.

The presence of early intervention in HIV-exposed infants is essential to the reduction of the MTCT of the virus. The findings of this review showed that HIV-exposed infants enrolled in ART care lately (> 6 weeks of age) had about seven times and those who did not receive NVP prophylaxis at all had about fourteen times higher odds of acquiring HIV from their mothers than their counterparts. This finding is in line with the review conducted in Ethiopia and India, which found that HIV-exposed infants who were enrolled in care late and had no ARV prophylaxis were more likely to get HIV infection [17, 18, 51]. The result is also consistent with WHO reports that without any maternal and/or child PMTCT intervention, 20 to 45% of infants will be infected by HIV [8, 54]. This could be due to the benefits of ARV drugs in reducing maternal viral load and acting as pre- and post-exposure prophylaxis against HIV infection in infants, thereby reducing the risk of HIV transmission from mother to child [16].

This systematic review and meta-analysis used studies conducted among women who enrolled in PMTCT programs only after Option B + implementation in Ethiopia and therefore provided the national magnitude of the MTCT of HIV for existing programs. However, there were some limitations to this review. First, this review included cross-sectional studies, which made it difficult to predict the cause-and-effect relationship for MTCT of HIV. Second, the meta-analysis of the risk factors was based on unadjusted odds ratios, and hence the results in this review might have been affected by confounding factors. On the other hand, there is high heterogeneity between some primary studies, although reported in subgroup analysis, due to the use of secondary data, which may be affected by its incompleteness and inaccuracy. Finally, the use of different types of HIV test kits to ascertain HIV infection status at different periods might affect diagnostic accuracy for HIV positivity among exposed infants.

## Conclusion

Ethiopia achieved the WHO target for MTCT of HIV after implementing the Option B + PMTCT program. However, mothers with poor adherence, clinical stage 2 or above, and lower CD4 counts were more likely to transmit HIV to their infants. Besides, risks were higher among infants born at home, mixed feeding practices, recently enrolled in care, and poorly adhered to nevirapine prophylaxis. Strengthening early PMTCT intervention, counseling on adherence, and infant feeding practices is recommended to tackle MTCT in Ethiopia.

### Electronic supplementary material

Below is the link to the electronic supplementary material.

Supplementary Material 1

Supplementary Material 2

Supplementary Material 3

Supplementary Material 4

Supplementary Material 5

## Data Availability

All data generated or analyzed during this study is included in this article and its supplementary information files.

## Abbreviations

AIDS: Acquired Immunodeficiency Syndrome
ARV: Antiretroviral
AJOL: African Journals Online
OR: Odds Ratio
ART: Antiretroviral Therapy; ARV: Antiretroviral
CI: Confidence Interval
HIV: Human Immunodeficiency Virus
JBI: Joanna Briggs Institute
MTCT: Mother-to-Child Transmission
PMTCT: Prevention of Mother-to-Child Transmission
