# Supplementary material for: Magnitude and risk factors of mother-to-child transmission of HIV among HIV-exposed infants after Option B+ implementation in Ethiopia: a systematic review and meta-analysis
Source: AIDS Res Ther. 2024 Jun 7;21:39. doi: 10.1186/s12981-024-00623-6 (PMC11157738; doi:10.1186/s12981-024-00623-6)
Supplement: Supplementary file 1 — Supplementary Material 1 [file 12981_2024_623_MOESM1_ESM.docx]

**PubMed search example**

(((((((((((((((Associated factors) OR Determinants) OR Predictors) OR Prevalence[MeSH Terms]) OR Incidence[MeSH Terms]) AND Infant[MeSH Terms]) OR Infant, Newborn[MeSH Terms]) AND HIV[MeSH Terms]) OR HIV Testing[MeSH Terms]) OR HIV Seroprevalence[MeSH Terms]) OR HIV Seropositivity[MeSH Terms]) OR HIV Infections[MeSH Terms]) OR Acquired Immunodeficiency Syndrome[MeSH Terms]) OR Infectious Disease Transmission, Vertical[MeSH Terms]) OR vertical transmission) AND Ethiopia [MeSH Terms].
