# Supplementary material for: Magnitude and risk factors of mother-to-child transmission of HIV among HIV-exposed infants after Option B+ implementation in Ethiopia: a systematic review and meta-analysis
Source: AIDS Res Ther. 2024 Jun 7;21:39. doi: 10.1186/s12981-024-00623-6 (PMC11157738; doi:10.1186/s12981-024-00623-6)
Supplement: Supplementary file 5 — Supplementary Material 5 [file 12981_2024_623_MOESM5_ESM.docx]

Figure 1. Forest plot that show pooled OR for home delivery

Figure 2. Forest plot that show pooled OR for maternal ART intervention

Figure 3. Forest plot that show pooled OR for poor ART adherence

Figure 4. Forest plot that show pooled OR for women who initiated ART during pregnancy

Figure 5. Forest plot that show pooled OR for WHO stage 2 and above

Figure 6. Forest plot that show pooled OR CD4 count below 350 count/mm3

Figure 7. Forest plot that show pooled OR for low/no male partner involvement

Figure 8. Forest plot that show pooled OR for negative partner status

Figure 9. Forest plot that show pooled OR for mixed feeding practice

Figure 10. Forest plot that show pooled OR for infants enrolled to care after 6 weeks of life

Figure 11. Forest plot that show pooled OR for infants no received NVP prophylaxis

Figure 12. Forest plot that show pooled OR for poor NVP adherence
